# Dissecting Bone Marrow Fibrosis in Philadelphia‐Negative Myeloproliferative Neoplasms: Multiple Roads Lead to Rome

**DOI:** 10.1002/hon.70247

**Published:** 2026-09-07

**Authors:** Mariagiovanna Ballato, Vincenzo Fiorentino, Gabriele Ricciardi, Pietro Tralongo, Emanuela Germanà, Alessandro Allegra, Fabio Stagno, Guido Fadda, Maurizio Martini

**Affiliations:** ^1^ Department of Biomedical, Dental, Morphological and Functional Imaging Sciences University of Messina Messina Italy; ^2^ Department of Human Pathology of Adult and Developmental Age “Gaetano Barresi” University of Messina Messina Italy

**Keywords:** aberrant megakaryocytes, bone marrow fibrosis, bone marrow microenvironment, fibrotic mechanisms, mutational landscape, Philadelphia‐negative myeloproliferative neoplasms

## Abstract

Philadelphia‐negative myeloproliferative neoplasms (MPN), a spectrum of blood malignancies characterized by uncontrolled myeloid proliferation, persist as a key challenge in onco‐hematology due to their intricate molecular background, heterogeneous clinical phenotypes, and variable prognoses. Although generally accepted as indolent disorders, a considerable fraction of MPN patients exhibit progressive, and sometimes accelerated, bone marrow fibrosis, reflecting a critical shift toward a more debilitating condition marked by an increased symptom burden. As marrow scarring advances, hematopoietic capacity declines, severely compromising both patients' quality of life and survival. Increasing evidence highlights the crucial involvement of multiple players and mechanisms orchestrating marrow fibrotic transformation, thereby reflecting the multifaceted nature of this pathogenetic process. In this review, we untangle the pro‐fibrotic programs underlying fibrosis progression in MPN, critically analyzing the available scientific literature. We explore the potential impact of the mutational landscape on the fibrotic transformation risk and examine the role of dysfunctional megakaryocytes as principal catalysts of fibrotic remodeling. Additionally, we provide insight into the influence of the bone marrow microenvironment, examining the contribution of both soluble factors and direct cell‐cell communication in sustaining and exacerbating the fibrotic signature. The urgent need for earlier diagnoses, tailored prognoses, and more effective therapeutic strategies stresses the importance of investigating the pathological basis of fibrotic progression from a deeper perspective.

## 1 Background

Philadelphia‐negative myeloproliferative neoplasm (MPN) represents an umbrella term for a heterogeneous collection of distinct yet closely related disorders that specifically affect the hematopoietic compartment and ordinarily follow a chronic clinical course. The natural history of MPN begins with the oncogenic transformation of a hematopoietic stem/progenitor cell following the acquisition of somatic driver mutations. This malignant reprogramming initiates and fuels the dominant expansion of the MPN clone, which typically results in abnormally high counts of circulating mature myeloid cell populations (i.e., erythrocytosis and/or thrombocytosis and/or leukocytosis) [1, 2, 3]. Negative for the reciprocal translocation t(9; 22) (q34; q11), the MPN spectrum includes three major pathological entities—polycythemia vera (PV), essential thrombocythemia (ET), and primary myelofibrosis (PMF) in both its pre‐fibrotic/early (pre‐PMF) and full‐blown stages (overt‐PMF)—as well as other, less common, hematologic disorders, such as chronic neutrophilic leukemia, chronic eosinophilic leukemia, not otherwise specified, and unclassifiable MPN [4, 5]. Specifically, PV, ET, and PMF—the classical MPN—will be the cornerstones of the present discussion [6].

Prognostication in MPN patients has proven to be particularly challenging. Despite their reputation as slow‐progressing malignancies, with survival times spanning years or even decades in the most optimistic scenarios, this indolent behavior is often disrupted by the emergence of severe complications, including evolution toward fibrotic stages marked by progressive marrow failure [7, 8, 9]. Bone marrow fibrosis (BMF) is the quintessential feature of PMF, representing a central diagnostic criterion. However, all MPN forms can develop, in due course, BMF, since both PV and ET often exhibit an intrinsic predisposition to evolve into secondary myelofibrosis—referred to as post‐PV MF and post‐ET MF, respectively— with a subgroup of patients experiencing a rapid disease progression [10, 11].

Far from being a mere architectural scaffold, the extracellular matrix (ECM) is a dynamic milieu that undergoes abnormal remodeling in various pathological settings, altering both its tissue‐specific composition and structural organization [12]. Specifically, BMF can be described as an increased deposition of stromal fibers within the marrow cavity that, over time, disrupts its ability to sustain effective hematopoiesis. Consequently, MPN patients may develop cytopenias, splenomegaly, extramedullary hematopoiesis, and a corollary of constitutional symptoms. Although other disease states (lymphomas, multiple myeloma, metastatic solid tumors, as well as certain infectious or autoimmune disorders) can be frequently associated with a scarred marrow, a growing body of research indicates that fibrosis in MPN is driven by distinctive molecular signatures, specific cellular actors, and characteristic microenvironmental cues [13, 14, 15].

However, despite ongoing efforts in onco‐hematology, the specific underpinnings of MPN‐related fibrotic evolution are only partially decoded, remaining an open question. This gap in knowledge severely limits the design of effective therapies capable of preventing or at least ameliorating BMF. From a clinical perspective, allogeneic hematopoietic cell transplant still portrays the only alternative for long‐term remission; however, it is not a suitable option for the majority of cases [16]. A better understanding of MPN biology—including the mechanisms underlying fibrosis and the contributors to its exacerbation in a specific patient subset—could pave the way for proper patient stratification and concrete improvements in outcomes.

Taken together, these considerations provided the core rationale for the realization of the present article: a narrative literature review aimed at comprehensively exploring the available evidence on the BMF pathogenesis in the setting of MPN. Specifically, we investigate the potential links between fibrotic evolution and the genetic background underpinning MPN, evaluate the contribution of aberrant megakaryocytes in promoting marrow scarring, and scrutinize the role of both direct and indirect interactions between the MPN clone and the surrounding niche in fostering aberrant fibrotic remodeling.

## 2 Genetic Alterations in MPNs and Their Impact on BMF Risk

The molecular pathogenesis of MPN has remained poorly understood for decades. However, more sophisticated tumor profiling techniques, especially high‐throughput Next Generation Sequencing (NGS) and microarray analyses, have profoundly advanced our comprehension of the disease, unveiling a dense molecular [17, 18, 19].

### 2.1 Driver Mutations

Approximately 80%–90% of MPN patients harbor somatic gain‐of‐function mutations in one of three driver genes, *Janus kinase‐2* (*JAK2*), *Calreticulin* (*CALR*), and *Thrombopoietin receptor* (*MPL* or *TPOR*), which directly or indirectly induce constitutive activation of the Janus Kinase/signal transducer and activator of transcription (JAK/STAT) signaling pathway [17]. Although these mutations typically arise in a mutually exclusive manner, rare cases of co‐occurrence has also been reported [20].

JAKs constitute a family of four cytoplasmic tyrosine kinases (JAK1, JAK2, JAK3, and TYK2) canonically activated when cytokines, growth factors, or hormones bind to specific receptors, including the erythropoietin, thrombopoietin, and granulocyte‐colony stimulating factor receptors. This interaction triggers a membrane‐to‐nucleus signaling cascade, culminating in the phosphorylation, dimerization and nuclear translocation of STAT transcription factors, modulating gene expression. The JAK/STAT axis tightly regulates crucial biological processes, such as cell fate, immune responses, and DNA repair, in a context‐dependent manner [21, 22].

Crucially, inappropriate activation of the JAK/STAT pathway promotes both proliferation and resilience of the MPN clone. In addition, current evidence suggests that it may contribute to the characteristic fibrotic evolution seen in MPN through multiple mechanisms, which remain partly nebulous. This signaling may act directly on the bone marrow stromal compartment, promoting the differentiation of bone marrow‐derived mesenchymal stromal cells (BM‐MSCs) into αSMA‐positive myofibroblasts with altered ECM composition and increased production of both fibronectin‐1 and the profibrotic matrix metalloproteinases‐9. Indirectly, it may support BMF by fueling a cytokine storm that perpetuates a highly inflammatory environment, a crucial element for disease progression [10, 23]. Supporting this, pan‐hematopoietic knockout of *STAT3* in a mouse model carrying the *MPL*W515L mutation resulted in decreased pro‐inflammatory cytokines secretion and reduced reticulin fiber deposition, as well as improved clinical outcomes. Conversely, *STAT3* deletion confined exclusively to the malignant clone failed to produce comparable effects. Therefore, the JAK/STAT pathway—and STAT3 in particular—functions as a central node in the crosstalk between clonal expansion and inflammation, mediating cytokine release from both tumor and non‐tumor cells [24].

### 2.2 Allele Burden

Expanding beyond signaling alterations, quantitative changes in the JAK2, MPL, and CALR mutant allele burden (the ratio of mutant to total alleles) have been investigated as key determinants of disease evolution and fibrotic progression in MPN. In a cohort of 407 MPN cases higher *JAK2* (V617F and exon 12 mutations) and *CALR* (type 1 and type 2) allele burdens were significantly associated with increased reticulin deposition. Particularly, patients with overt fibrosis (grade ≥ 2) exhibited considerably higher *JAK2* or *CALR* allele burdens and poorer prognoses compared to those with fibrosis grade < 2 (*p* < 0.001) [25]. These findings reinforce previous results from an Italian study which evaluated the prognostic relevance of mutant allele burden in 1185 patients with PV or ET, indicating that the risk of progression to secondary MF rises with increasing *JAK2*V617F allele burden. Furthermore, *CALR* (type 1 or type 2) mutant allele burden was significantly higher in cases with post‐ET MF compared to those with ET [26]. A third study involving 490 MPN patients investigated the potential correlation between marrow histology, including fibrosis grade, and variations in both *JAK2*V617F and *MPL*W515L allele burden. Long‐term follow‐up data showed that while the *JAK2*V617F allele burden remained largely stable during progression from the pre‐fibrotic to the fibrotic stage, *MPL*W515L allele burden raised in tandem with the fibrotic transformation of the bone marrow [27].

### 2.3 Non‐Driver Mutations

**FIGURE 1:**
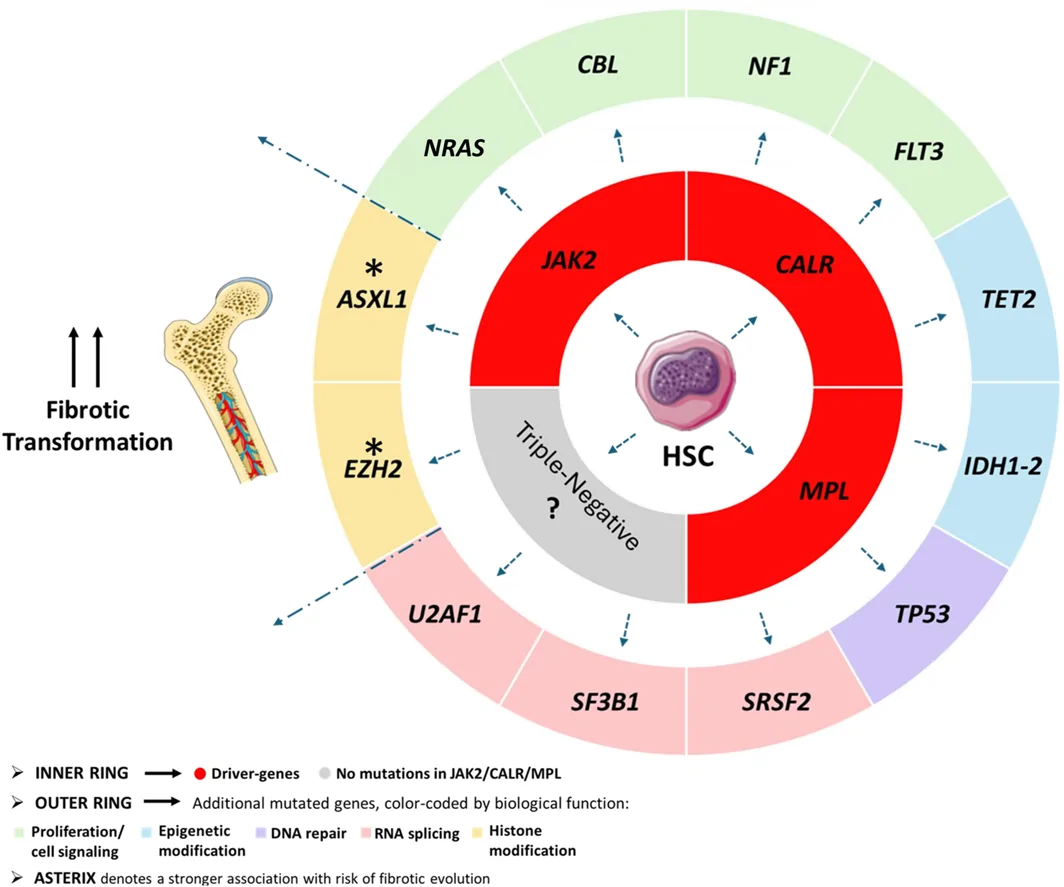
Hierarchical representation of mutated genes in Philadelphia‐negative myeloproliferative neoplasms (MPN). This schematic figure illustrates the mutational landscape associated with MPN etiology and progression, including their evolution toward bone marrow fibrosis. ASXL1, Additional sex combs‐like 1; CALR, calreticulin; CBL, Casitas B‐lineage lymphoma proto‐oncogene; EZH2, enhancer of zeste 2 polycomb repressive complex 2 subunit; FLT3, Fms‐like tyrosine kinase 3; HSC, Hematopoietic stem cell; IDH1/2, Isocitrate dehydrogenase 1/2; JAK2, Janus kinase 2; MPL, thrombopoietin receptor; NF1, neurofibromin 1; NRAS, neuroblastoma Kirsten rat sarcoma viral oncogene homolog; SF3B1, splicing factor 3b subunit 1; TET2, Tet methylcytosine dioxygenase 2; TP53, tumor protein p53; SRSF2, serine and arginine rich splicing factor 2; U2AF1, U2 small nuclear RNA auxiliary factor 1. Some elements of the figure were drawn using pictures from Servier Medical Art (https://smart.servier.com (accessed on 10 October 2025)).

Intriguingly, over 50% of MPN patients exhibit additional genomic aberrations in non‐driver genes implicated in a wide variety of cellular contexts, including epigenetic regulation (*TET2*, *IDH1/2*), chromatin structure remodeling (*ASXL1*, *EZH2*), DNA damage response (*TP53*), splicing mechanisms (SRSF2, SF3B1, U2AF1), and signal transduction or cell proliferation (*NRAS*, *CBL*, *NF1*, *FLT3*) [28, 29, 30, 31, 32, 33, 34, 35, 36]. These genetic alterations may coexist with driver mutations or occur independently, as documented in so‐called “triple‐negative” MPN patients, and their timing and order of acquisition may shape the resulting MPN phenotype [37, 38, 39]. Overall, this increasingly intricate genetic scenario (Figure 1) exhibits marked inter‐patient heterogeneity, variably impacting both clinical presentation and prognosis. Of note, several studies have consistently linked *ASXL1* and *EZH2* mutations with accelerated BMF and poorer prognosis in MPN cases [29, 40, 41, 42, 43, 44, 45]. However, the biological and molecular basis underlying these associations remains unclear, requiring further investigation. Preliminary data suggest that mutated *ASXL1* may promote fibrotic transformation by inducing the generation of neoplastic monocyte‐derived fibrocytes and amplifying monocyte/macrophage‐mediated inflammation through the EGR1‐TNFα pathway in both engineered mice and MPN patients [43, 46]. Along the same lines, *EZH2* loss has been observed to synergize with the *JAK2*V617F mutation in murine models, contributing to rapid fibrotic progression. From a mechanistic perspective, *EZH2* deficiency is thought to abnormally activate numerous oncogenes, notably *Hmga2*, which increases TGF‐β1 and Cxcl12 pathway activity, thus promoting collagen deposition in the marrow microenvironment [47, 48].

However, the available information on the mutational background of HSCs alone does not completely explain the onset of MPN or the evolution of the disease toward fibrotic stages, suggesting that additional mechanisms and actors play pivotal roles in disease pathogenesis and progression. In recent years, novel fibrosis‐associated cellular determinants and soluble mediators, particularly pro‐inflammatory ones, have gained increasing attention and will be explored thoroughly in the following sections.

## 3 Pro‐Fibrotic Role of Megakaryocytes

Mature megakaryocytes (MKs) are large, polyploid cells with a multilobed nucleus and granule‐rich cytoplasm. Predominantly residing in the bone marrow, MKs can also be found in extramedullary sites such as the lungs, liver, and spleen. This cellular population originates from HSCs via megakaryopoiesis, a meticulously regulated process primarily guided by thrombopoietin (TPO). Functionally, MKs are highly specialized cells that predominantly work as “platelet factories”, participating in coagulation, tissue repair, and immune responses. More recently, MKs have emerged as core components of ancillary processes involving the bone marrow microenvironment, such as ECM remodeling, neovascularization, and the regulation of HSC behavior, in both physiological and disease‐related scenarios [49, 50, 51].

Although long debated, mounting evidence currently supports the pivotal role of dysfunctional MKs in orchestrating the fibrotic progression seen in a significant proportion of MPN patients [52]. Importantly, in transgenic mouse models, aberrant activation of JAK/STAT signaling restricted to MKs appears sufficient to sustain a myeloproliferative disease in vivo, also promoting bone marrow scarring [53]. Consistently, in mice, reduced levels of Gata1, a key regulator of normal erythropoiesis and megakaryopoiesis, leads to MK abnormalities and progressive collagen deposition in the bone marrow, recapitulating human PMF [54]. Similarly, TPO‐driven models support a causal role for dysregulated megakaryopoiesis, as TPO‐overexpressing mice develop BMF and osteosclerosis, along with increased MK counts and anemia [55]. In line with these findings, transplantation of bone marrow cells engineered to overexpress TPO leads to improper megakaryocyte expansion, defective differentiation, apoptosis resistance, and severe BMF [56, 57].

**FIGURE 2:**
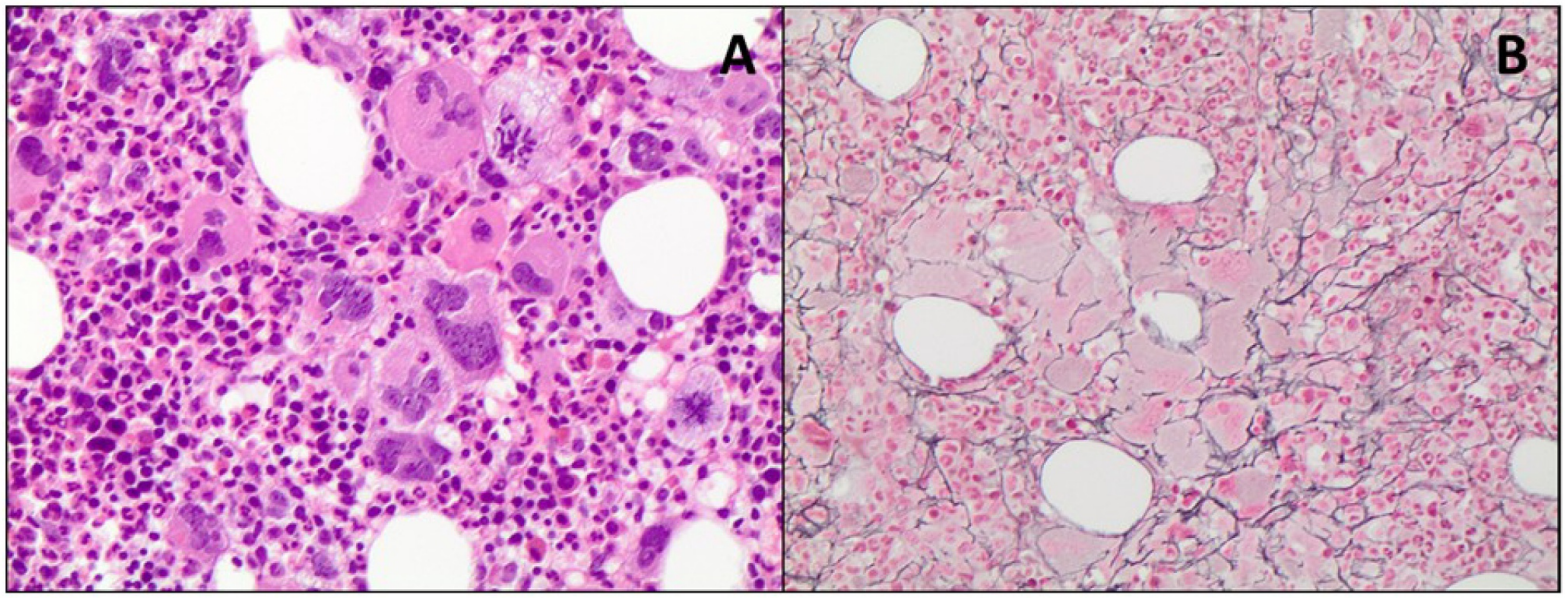
Representative bone marrow sections from a case of prefibrotic myelofibrosis showing clustered atypical megakaryocytes and perimegakaryocytic reticulin reinforcement. (A) Hematoxylin–eosin staining (× 25) reveals prominent clusters of markedly atypical megakaryocytes with enlarged, hyperchromatic, and irregularly folded nuclei, often exhibiting bulbous nuclear lobulation and dense eosinophilic cytoplasm. At the center, a megakaryocyte displaying emperipolesis is evident, containing an intact granulocytic precursor within its cytoplasm. (B) The corresponding reticulin stain (× 25) demonstrates conspicuous reinforcement of the reticulin framework surrounding both clusters and individual megakaryocytes, outlining early perimegakaryocytic stromal remodeling typical of megakaryocytic activation.

Numerical and morphological alterations in MKs represent a common denominator across different MPN entities. In the bone marrow, where MKs are normally scarce, a marked megakaryocytic hyperplasia is observed, accompanied by pleomorphism (larger cell size, nuclear atypia, higher nuclear: cytoplasmic ratio, and reduced polyploidy) together with scattered clustering [58, 59]. By retrospectively evaluating bone marrow biopsies from MPN patients, our group previously characterized a novel morphological parameter, named Megakaryocytic Activation (M‐ACT), as a potential predictor of fibrotic transformation and worse prognosis (Figure 2). M‐ACT, defined by the simultaneous presence of megakaryocytic emperipolesis, MKs clustering, and peculiar collagen fiber arrangements encircling MKs, might represent the morphological equivalent of molecular events theorized in multiple in vitro/in vivo experiments [56]. Additionally, the characteristic MKs abnormalities observed in MPN have provided a rationale for targeting Aurora Kinase A (AURKA), a key mitotic serine/threonine kinase hyperactivated downstream of oncogenic JAK/STAT signaling. AURKA inhibitors have shown therapeutic potential by stimulating MK polyploidization and differentiation, as well as mitigating BMF in mouse models of PMF [59].

There is growing evidence supporting the role of MKs as the main “inciters” of MPN‐related fibrogenesis. MKs are thought to exert much of their pathogenic activity by releasing a series of mediators, including interleukins (IL‐6, IL‐1β), interferons (IFNs), tumor necrosis factors (TNF‐α), chemokines (PF‐4), growth (TGF‐β, PDGF, FGF) and pro‐angiogenic factors (VEGF), all closely implicated in disease progression [60, 61].

## 4 Cellular Crosstalk Within Bone Marrow Microenvironment: Emerging Dynamics in Fibrosis Progression

The MPN clone, like a skilled puppeteer, profoundly manipulates the surrounding marrow microenvironment, establishing favorable conditions for disease progression and clonal resilience. The bone marrow microenvironment assumes a permissive phenotype, evolving into a sophisticated network that significantly contributes to MPN pathogenesis [62]. The dialog between aberrant HSCs and neighboring marrow cells involves both direct cell‐to‐cell contacts and the release of soluble mediators. However, their specific contribution to MPN‐associated BMF warrants further research efforts.

### 4.1 Soluble Signals

In the context of MPN, if genetic alterations may be “the spark that sets cancer in motion”, a chronic inflammatory milieu can act as “the oxygen that sustains and intensifies the flames of neoplasm”. Malignant HSCs proliferate uncontrollably, and together with their differentiated descendants, improperly secrete elevated levels of pro‐inflammatory mediators that impact the surrounding microenvironment. Within this inflammatory, disease‐permissive ecosystem, even non‐malignant cell populations become dysregulated and contribute to the inflammatory state by releasing additional cytokines, thereby sustaining disease progression. A vicious pathological thus emerges, in which the MPN clone and the inflammatory microenvironment reinforce each other [13].

**FIGURE 3:**
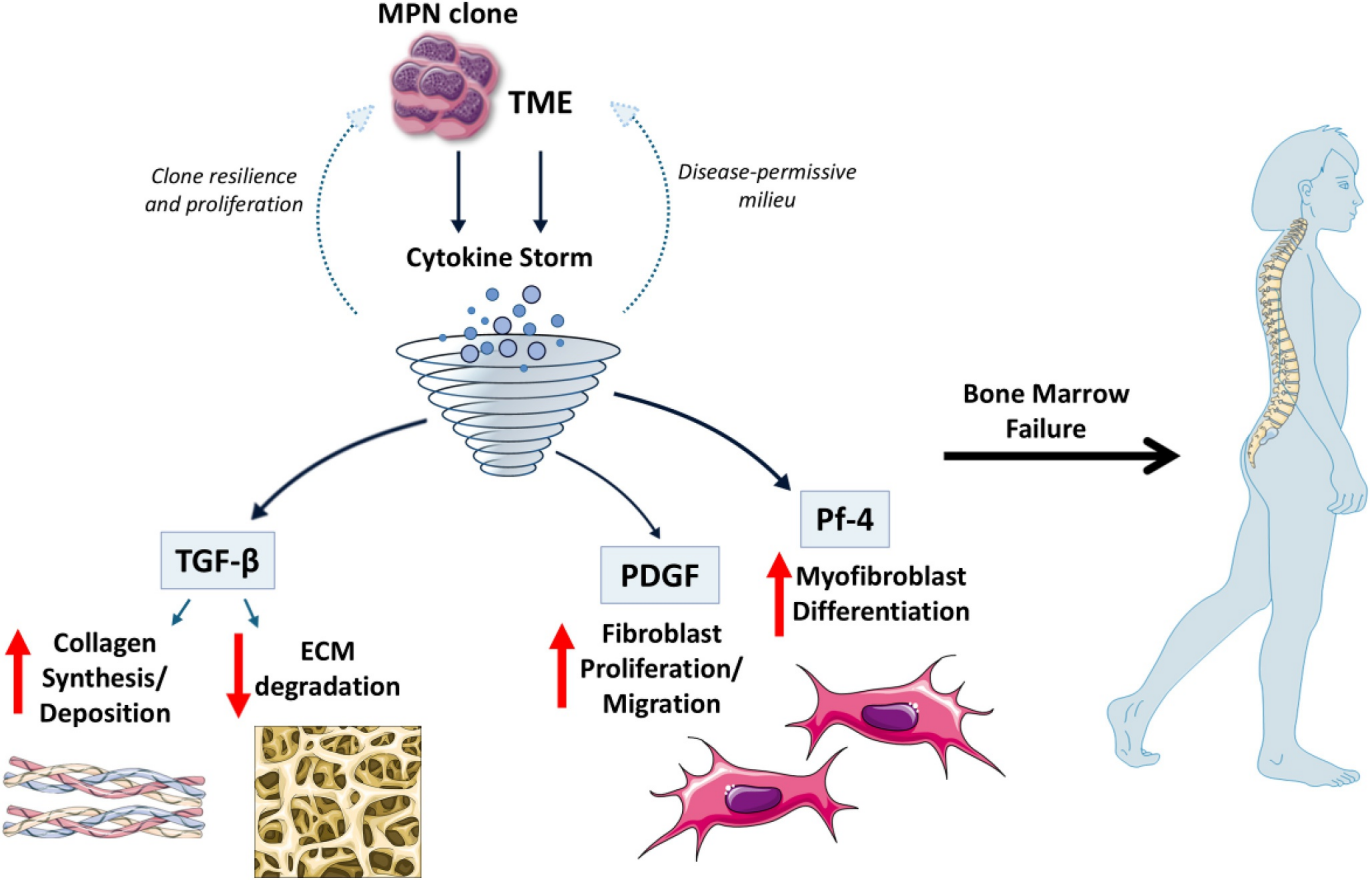
Key pro‐fibrotic mediators driving marrow scarring in Philadelphia‐negative myeloproliferative neoplasms (MPN). Both the MPN clone and the bone marrow microenvironment fuel a cytokine storm, leading to chronic inflammation, which, in a vicious circle, promotes the survival of malignant hematopoietic stem cells and reshapes the marrow niche into a permissive ecosystem. Among the pro‐inflammatory factors, TGF‐β, PDGF, and PF‐4 exert pro‐fibrotic effects at multiple levels, ultimately contributing to bone marrow failure. TME, tumor microenvironment; ECM, extracellular matrix; TGF‐β, transforming growth factor beta; PDGF, platelet‐derived growth factor; PF4, platelet factor 4. This figure was created using Servier Medical Art (https://smart.servier.com, accessed on 10 October 2025) and NIH Bioart (https://bioart.niaid.nih.gov, accessed on 10 October 2025).

Multiple lines of evidence indicate that MPN patients, especially in advanced stages, commonly exhibit dysregulated cytokine and chemokine production, reflected in higher circulating levels of these mediators in the bloodstream [63, 64]. Furthermore, large‐scale cytokine profiling analyses support the existence of specific inflammatory cytokine signatures depending on the MPN subtype and its severity [65]. The pathogenic relevance of cytokine imbalance and its causal link to MPN progression have increasingly been recognized. In particular, a pro‐inflammatory state has been associated with bone marrow fibrotic transformation, osteosclerosis, angiogenesis, constitutional symptoms, and an amplified risk of thrombotic events [63, 66]. The key soluble mediators most directly involved in MPN‐associated BMF will be discussed below and illustrated in Figure 3.

#### 4.1.1 TGF‐β

The transforming growth factors beta (TGF‐β) family, comprising three isoforms: TGF‐β1, TGF‐ β2, and TGF‐β3, represents a small class of cytokines that play multiple physiological and pathological roles in the human body. Among TGF‐β isoforms, TGF‐β1 appears most frequently upregulated in various tumor types, including blood cancers [67]. In MPN, numerous studies have indicated TGF‐β as “the master cytokine” governing fibrotic responses, albeit not the only contributor [68]. TGF‐β exerts dichotomous effects: it can actively promote the biosynthesis and deposition of type I and type III collagen, proteoglycans, and fibronectin, while simultaneously inhibiting proteolysis, thereby limiting ECM degradation. It can also induce fibroblast chemotaxis [69, 70].

Pioneering experiments provided the first clinical evidence in the 1990s, revealing elevated intraplatelet and serum TGF‐β levels in MPN patients with BMF, compared to controls [71, 72]. Approximately 80% of systemic TGF‐β was activated and attributable to isoform—β1. TGF‐β1 overexpression was also detected in fibrotic bone marrow biopsies through both in situ hybridization and immunohistochemistry [72].

In *MPL*W515L and *JAK2*V617F mouse models, blocking TGF‐β signaling in Osx‐Cre‐targeted MSCs arrested fibrotic transformation, without restoring the altered hematopoietic niche. In the same study, the use of a c‐Jun N‐terminal kinase inhibitor prevented myelofibrosis development in *MPL*W515L mice, suggesting that the JNK‐mediated TGF‐ β pathway occupies a key position in fibrotic progression [73]. Regarding therapeutic prospects, the efficacy of numerous approaches (monoclonal antibodies, ligand traps, vaccines, receptor kinase antagonists, and antisense oligonucleotides) against TGF‐β axis has been investigated in different tumor contexts [74]. A phase 1b, multicenter clinical study tested AVID200, a specific TGF‐β 1/3 trap, in 21 patients with MF. Preliminary findings demonstrated low toxicity, increased platelet count in most patients, and decreased TGF‐β1 and pSMAD2 in plasma and MF cells, respectively. Further investigation is required to evaluate its efficacy in attenuating BMF, especially in combination with other therapeutic agents [75]. Meanwhile, a TGF‐β receptor I kinase inhibitor showed reduced collagen deposition and mitigated fibrotic phenotype in preclinical experiments, raising hopes about the therapeutic benefits of targeting this signaling pathway [76].

#### 4.1.2 PDGF

The five well‐characterized platelet‐derived growth factor (PDGF) isoforms (PDGF‐AA, ‐AB, ‐BB, ‐CC, and ‐DD) mediate numerous biological activities, including tissue regeneration, cellular proliferation, differentiation, and migration, through the activation of PDGF receptors (PDGFRs, PDGFR‐α and PDGFR‐β). As PDGF is a potent stimulator of mesenchymal cell replication and migration, as well as collagen deposition, its potential roles in organ fibrosis have been extensively investigated [77, 78]. Disrupted PDGF/PDGFR signaling networks have been linked to the pathogenesis of renal, hepatic, pulmonary, and cardiac fibrosis, in addition to tumor‐associated fibrosis, especially in MPN [78].

Real‐time reverse‐transcription polymerase chain reaction (qRT‐PCR) assays conducted on bone marrow cells from patients with idiopathic myelofibrosis showed overexpression of all PDGF isoforms and PDGFR‐α in advanced myelofibrosis compared to controls [79]. With a different focus, Bedekovics and collaborators evaluated PDGFRβ involvement in MF using immunohistochemistry analysis of 60 bone marrow biopsies. PDGFR subunits exhibited differential expression patterns across parenchymal cell populations: MKs, endosteal, and endothelial cells express PDGFR‐α specifically, while PDGFR‐β was markedly expressed only by activated fibroblasts. PDGFRβ upregulation correlated with fibrotic progression [80]. Méhes et al. observed similar findings, suggesting increased PDGFR‐β immunoreactivity as a valuable predictor of disease progression [81]. While Baglin et al. found lower platelet PDGF content in MPN patients compared to controls, an American group, using ELISA, revealed elevated plasma and urine PDGF levels in ET (5.5 +/− 1.5 μg/L for plasma; 11.4 +/− 2.2 μg/L for urine) and MF (6.2 +/− 2.0 μg/L for plasma; 7.8 +/− 2.4 μg/L for urine), but not in PV cases [82, 83]. In 2014, Pourcelot et al. added a further piece to the puzzle, revealing differential expression of PDGF and other MKs‐released mediators, depending on the MPN subtype, suggesting cytokine assessment as a possible tool for differential diagnosis [84]. In a subsequent German study, Gata1^low^ mice exhibited increased protein expression of PDGFRβ and its ligand PDGF‐B. In situ proximity ligation assay also revealed close spatial proximity between PDGFR‐β and PDGF‐B in overtly fibrotic bone marrow [85]. Moreover, imatinib‐mediated PDGFR‐α inactivation strongly reduced proliferation of leptin receptor‐positive mesenchymal stromal cells and BMF in TPO‐overexpressing mice [86].

#### 4.1.3 PF4/CXCL4

Platelet factor 4 (PF4), otherwise known as CXCL4, is a heparin‐binding protein with a molecular weight of approximately 8 kDa and is a specific member of the ELR‐negative CXC chemokines. MKs are the main source of PF4, which is predominantly stored in platelets' *α*‐granules. PF4 appears to play a strategic role in a plethora of biological functions, such as coagulation, wound healing, neovascularization, and modulation of immune cell behavior [87]. However, accumulating evidence also argues for PF4's contribution to dysregulated processes, including tumor growth and fibrotic tissue remodeling.

Affandi et al. employing different murine models of skin, lung and cardiac fibrosis, detected increased PF4 levels both in tissues and plasma compared with controls. Furthermore, human PF4 overexpression exacerbated, whereas PF4 blockade mitigated, bleomycin‐induced skin fibrosis in mice [88]. Similarly, PF4 inhibition reduced liver fibrosis in models of chronic liver allograft dysfunction [89]. Recently, PF4 has emerged as a crucial *fil rouge* connecting inflammation to fibrosis in the MPN scenario. In 1984, Burstein and colleagues have already measured PF4 levels in plasma, platelets, and urine of 49 patients with myeloproliferative neoplasms, hypothesizing that mediators released from aberrant MKs, including PF4, promoted excessive fibroblast proliferation. While increased urinary PF4 was associated with MF, serum and platelet levels showed no correlation with BMF [90]. Conversely, mass spectrometry‐based proteomics in rat models of myelofibrosis by Capitanio et al. indicated higher PF4 levels in plasma and BM fluids. PF4 overexpression in MKs/platelets and higher bone marrow levels were also detected in MPN patients but restricted to overt fibrosis cases. The same study demonstrated that surface glycosaminoglycans can mediate rapid uptake of PF4 by stromal cells, thus stimulating myofibroblast differentiation [91]. Consistent with these findings, Gleitz's laboratory, using mouse models of PMF, observed that genetic inactivation of *PF4* alone reduced stromal stimulation and BMF but did not fully reconstitute normal marrow architecture; more drastic decreases were seen in megakaryocytic dysplasia, inflammation, and JAK/STAT signaling in both stromal cells and MKs. Overall, these data suggest that multiple mediators cooperate in MPN‐specific fibrogenesis, strongly encouraging the design of multi‐targeted therapies including PF4 [61].

### 4.2 Direct Cell‐Cell Interactions in BMF

The lively dialog between the MPN clone and the surrounding niche components orchestrates disease progression not only through the release of soluble mediators that promote fibrotic and inflammatory responses, but also via direct cell‐cell interactions. However, to date, only limited attention has been given to the dynamics of physical intercellular communication in MPN. A deeper understanding of these mechanisms and their role in fibrotic transformation could unveil hidden aspects of MPN pathogenesis and pave the way for future treatment approaches.

In support of this emerging concept, Zingariello and colleagues examined the fibrotic spleen of Gata1^low^ myelofibrotic mice by immunohistochemistry and immuno‐transmission electron‐microscopy analyses. The research group revealed a peculiar pathological interaction, called peripolesis, between aberrant megakaryocytes and activated fibrocytes, in which fibrocytes with their protrusions surround and penetrate the megakaryocyte, directly releasing collagen associated with TGF‐β. Megakaryocytes, embedded within collagen fibers, undergo electron‐dense para‐apoptosis, releasing TGF‐β and increasing its bioavailability in the surrounding microenvironment. The role of TGF‐β is central to fibrocyte activation. However, the adhesion receptor P‐selectin also plays a crucial part in mediating peripolesis and promoting fibrosis as its genetic ablation prevents myelofibrosis in Gata1^low^ mice, emphasizing the significance of direct cellular interactions [92]. P‐selectin, abnormally overexpressed by malignant megakaryocytes, has also been implicated in neutrophil emperipolesis with MKs, another dysfunctional interaction observed in MPN. This phenomenon directly fuels fibrosis by triggering the release of activated TGF‐β and fosters a dysregulated niche that supports MPN clone proliferation. Notably, preclinical studies have already shown that pharmacological inhibition of P‐selectin, in combination with ruxolitinib (JAK1/2 inhibitor), can restore a normal phenotype in Gata1^low^ mice with established MF, further reinforcing the role of cell‐cell contact [93].

Furthermore, direct interactions between the MPN clone and BM‐MSCs stimulates the overproduction of functionally altered osteoblasts, which accumulate as inflammatory myelofibrotic cells. This drives substantial endosteal BM niche remodeling that favors malignant HSC proliferation and aberrant ECM deposition at the expense of normal hematopoiesis. As demonstrated in chronic myeloid leukemia murine models, this phenomenon remains to be validated in in vivo MPN models [94]. In addition, the crosstalk between aberrant MKs and the vascular niche, physiologically modulated by adhesion molecules (VCAM‐1, PECAM‐1, and VE‐cadherin), MK membrane receptors, and other soluble factors, requires further investigation. The *JAK2*V617F mutation seems to dysregulate MK‐endothelial interplay, leading to vascular niche reshaping (increased vascular density, enhanced sinusoidal caliber, and a higher number of MKs along marrow sinusoids), which could contribute to thrombosis and HSC clonal expansion [95].

Moreover, the involvement of mechanical stimuli in fibrotic evolution has largely remained unexplored. Recently, Teodorescu et al. showed, employing fibroblast cell lines from PMF patients and TGF‐β receptor (TGFBR) 1 and 2 knock‐down PMF‐derived fibroblasts, that mechanical stress, through TGF‐β pathway, can promote fibroblast proliferation, migration, and increased collagen deposition [68].

**FIGURE 4:**
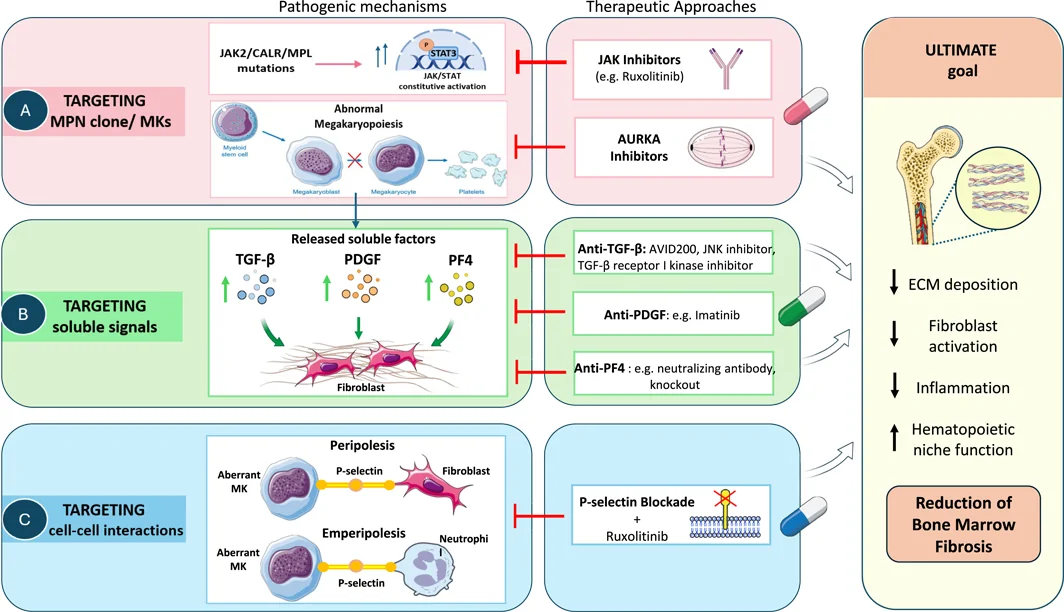
Multi‐level therapeutic strategies in MPN‐associated bone marrow fibrosis (BMF). The figure illustrates three complementary levels of intervention targeting the cellular and molecular mechanisms driving fibrotic progression, including extracellular matrix deposition, fibroblast activation, inflammation, and hematopoietic niche dysfunction. (A) Targeting the malignant clone through modulation of intracellular signaling pathways, such as JAK and Aurora kinase A. (B) Targeting profibrotic soluble mediators within the bone marrow microenvironment, including TGF‐β, PDGFR, and PF4. (C) Targeting direct cell–cell interactions involved in fibrotic niche remodeling through P‐selectin blockade, preventing pathological peripolesis and emperipolesis. The depicted approaches span clinically approved therapies and strategies currently under preclinical or clinical investigation. AURKA, Aurora kinase A; CALR, calreticulin; ECM, extracellular matrix; JAK2, Janus kinase 2; MKs, megakaryocytes; MPL, thrombopoietin receptor; MPN, Philadelphia‐negative myeloproliferative neoplasms; PDGF, platelet‐derived growth factor; PF4, platelet factor 4; TGF‐β, transforming growth factor beta. Some elements of the figure were drawn using pictures from Servier Medical Art (https://smart.servier.com (accessed on 20 June 2026)) and NIH Bioart (https://bioart.niaid.nih.gov, accessed on 20 June 2026).

Taken together, the available evidence suggests that MPN‐associated fibrogenesis is a multifactorial process, in which both direct cellular interactions and mechanical stimuli play underappreciated roles. In this framework, the diverse molecular, cellular, and microenvironmental determinants discussed delineate a multi‐tier map of therapeutic vulnerabilities across the bone marrow compartments, as summarized in Figure 4. These multiple intervention sites highlight the translational relevance of targeting the interconnected mechanisms driving bone marrow fibrosis in MPN, an emerging paradigm in modern patient care.

## 5 Conclusion

In MPN, bone marrow fibrotic transformation marks a crucial turning point, signaling an advanced stage of the disease characterized by impaired medullary function and a consequent negative impact on patients' clinical outcomes. Overall, a key concept emerging from the examined literature is the profound complexity underlying BMF pathogenesis, which continues to pose significant challenges for accurate patient stratification and the design of effective treatment strategies. Currently, MPN management remains largely limited to symptomatic relief, with only a minority of cases benefiting from bone marrow transplantation. This highlights the pressing requirement for multi‐targeted, patient‐tailored approaches that also pay attention to fibrotic dynamics. With ongoing research, BMF increasingly appears as a multifaceted process. Our current knowledge represents just a piece of a much larger puzzle. Only by delving deeper into this intricate network dominated by different molecular and cellular actors within a chronically inflammatory microenvironment, can we bring renewed hope to the care of MPN patients.

## Funding

The authors have nothing to report.

## Conflicts of Interest

The authors declare no conflicts of interest.

## Data Availability

Data sharing not applicable to this article as no datasets were generated or analysed during the current study.
